# Congenital Short-Bowel Syndrome Is Associated With a Novel Deletion Mutation in the *CLMP* Gene: Mutations in *CLMP* Caused CSBS

**DOI:** 10.3389/fped.2021.778859

**Published:** 2022-01-17

**Authors:** Fen-fen Ou, Ming-jie Li, Li-bin Mei, Xin-Zhu Lin, Yan-an Wu

**Affiliations:** ^1^Department of Neonatology, Women and Children's Hospital, School of Medicine, Xiamen University, Xiamen, China; ^2^Xiamen Key Laboratory of Perinatal-Neonatal Infection, Xiamen, China; ^3^Department of Clinical Laboratory, Xiang'an Hospital of Xiamen University, School of Medicine, Xiamen University, Xiamen, China

**Keywords:** congenital short bowel syndrome, CLMP gene, homozygous deletion, quantitative PCR, WES-whole-exome sequencing

## Abstract

**Objective:** To describe the clinical presentation and novel mutation in the coxsackie and adenovirus receptor-like membrane protein (*CLMP*) gene in a Chinese family with congenital short bowel syndrome (CSBS).

**Methods:** We collected clinical data from a Chinese family with inherited CSBS, and performed whole exon sequencing of the children and their parents. The pathogenic sites of candidate genes were targeted, and the detected exon deletions were verified by quantitative PCR.

**Results:** Two siblings in this family presented with bilious vomiting, and were diagnosed with CSBS on laparotomy. Two siblings and their parents underwent complete exome sequencing of the peripheral blood. Both children had *CLMP* gene exons 3–5 homozygous deletion mutation, while the parents had a heterozygous mutation.

**Conclusion:** This study identified a novel mutation of the *CLMP* gene in a Chinese family with CSBS. Identification of this mutation can help with genetic counseling and prenatal diagnosis of CSBS.

## Introduction

Congenital short bowel syndrome (CSBS) is a rare congenital disorder that is clinically characterized by a reduction in the length of the small intestine at birth. Compared with length of the small intestine of normal full-term infants (190–280 cm) (1, 2), the mean length in patients with CSBS is about 50 cm (3). The incidence of CSBS is <1 in one million births (4). The symptoms of CSBS are bilious vomiting, growth retardation, diarrhea, or gastrointestinal obstruction, most of which are accompanied by intestinal malrotation (5). CSBS is associated with a high mortality rate (6), but does not affect mental development (3, 7).

CSBS was first reported by Hamilton in 1969 (8). The exact pathogenesis is still unclear. Related hypotheses include interruption or delay of small intestine elongation and rotation due to lack of space in the umbilical cord during fetal development (8), defects in neurointestinal development (9), or vascular obstruction or ischemic damage to the primitive midgut (5). In 2012, Van et al. (10) found that knock-down of the coxsackie and adenovirus receptor-like membrane protein (*CLMP*) homologous gene in zebrafish resulted in significant shortening of the intestine and lack of goblet cells in the midgut, suggesting that *CLMP* is necessary for normal small intestinal development. It was speculated that recessive mutation of *CLMP* gene could lead to CSBS in humans.Homozygous and compound heterozygous mutations of *CLMP* in CSBS patients suggest that the inheritance pattern of the disease is autosomal recessive.

The *CLMP* gene is located on chromosome 11 (11q24.1), which encodes a transmembrane protein and co-localizes with the tight junction-related protein zonula occludens-1 as an adhesion molecule. It is one of the tight junction components of the epithelium (11), which is expressed in different tissues during embryonic development including the intestine (12). Since tight junction markers such as ZO-1 and its related nucleic acid binding proteins play an important role in cell proliferation (13, 14), and *CLMP* is co-localized with tight junction proteins, it is speculated that the loss of function of *CLMP* may affect the proliferation of small intestinal cells, leading to shortening of the small intestine at birth. Wang *et al*. examined the biopsies of five CSBS children with *CLMP* mutation and found chromatin clumps and pyknosis in the intermuscular nuclei of these patients, suggesting that *CLMP* gene mutation may affect the development of intestinal nerve plexus (15).In addition, a mutation in the filamin A (*FLNA*) gene was found in male patients with CSBS, which is related to the X-linked inheritance pattern (16).

We report a Chinese family with CSBS and analyzed their clinical features, treatment, and prognosis. The new homozygous deletion mutation of *CLMP* gene exons 3–5 was identified as the cause of CSBS in this family by using whole exon sequencing (WES) and quantitative PCR (qPCR).

## Patients and Methods

### Patients

**Figure 1:**
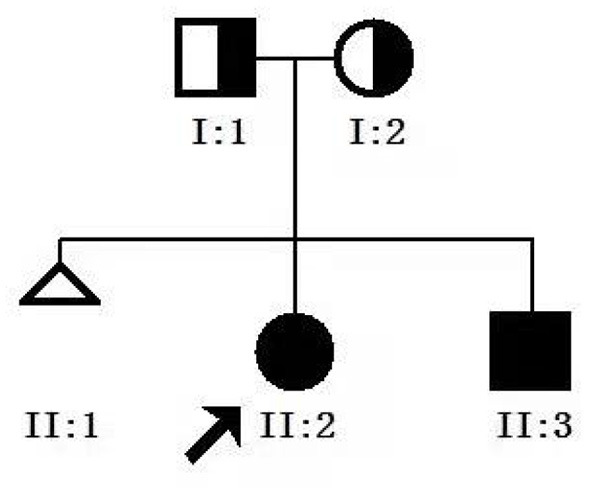
Genetic pedigree map for the proband. **I:1**, father; **I:2**, mother; **II.2**, proband; **II.3**, younger brother.

**Figure 2:**
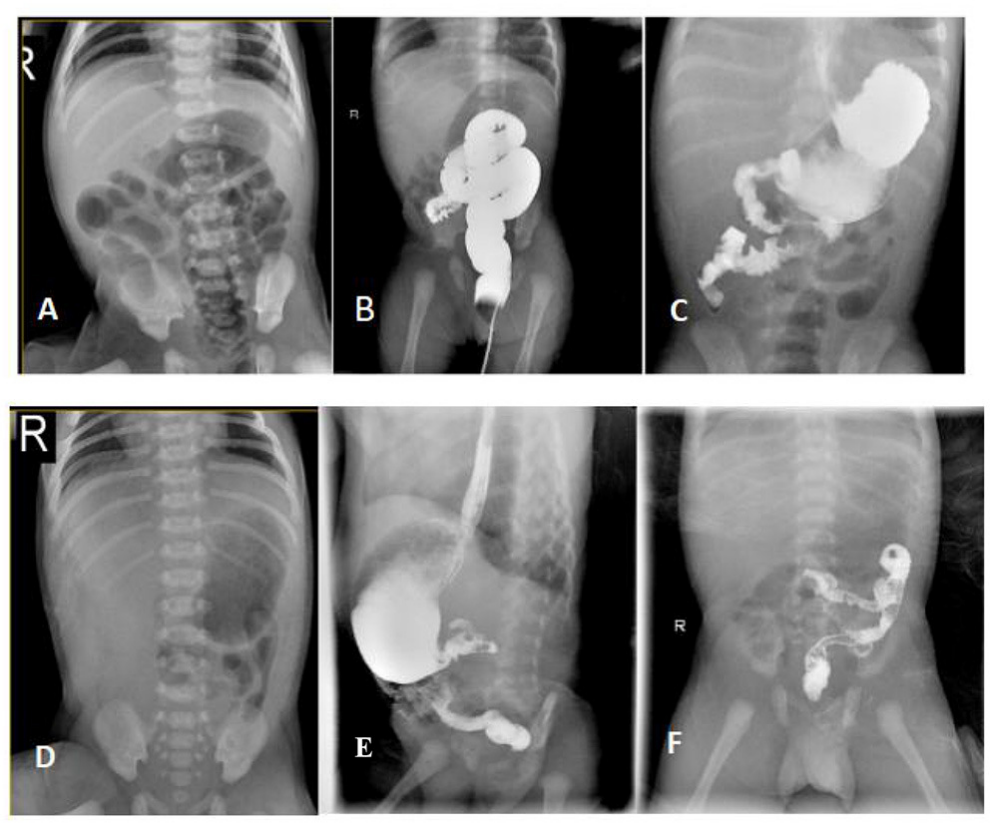
Radiological findings of the patients with CSBS. [The proband] **(A)** X-ray abdomen showing the gas-filled stomach and the intestinal loops; **(B)** Lower gastrointestinal radiography showing the distribution of the colon predominantly in the center and on the left side of the abdomen; **(C)** UGI showing the upper part of the jejunum located on the right side of the abdomen in a spiral pattern. [The younger brother] **(D)** X-ray abdomen showing minimal gas in the stomach with no obvious gas shadows in the intestine. **(E,F)** UGI showing the ascending part of the duodenum and the upper part of the jejunum in a spiral pattern.

A full-term female baby (II:2) with a birth weight of 2,550 g presented with recurrent vomiting soon after birth (Figure 1). On physical examination, the girl's abdomen was flat and soft, without distention, and the bowel sounds were normal. Blood tests showed that serum potassium was 2.69 mmol/L. X-ray presented the gas-filled stomach and the intestinal loops (Figure 2A) while the colon is mainly located in the center and left side of the abdomen (Figure 2B). Other blood investigations were normal. An upper gastrointestinal series (UGI) revealed intestinal malrotation (Figure 2C). The patient underwent Ladd's operation. At operation, the pediatric surgeon found that the total length of her bowel was only 65 cm from the duodenum to the ileocecal valve. After the operation, the girl initially developed diarrhea during breastfeeding, but gradually developed intestinal tolerance after changing to hydrolyzed milk and continuous nasogastric tube infusion. Parenteral nutrition (PN) was administered for up to 4 months. Fortunately, the child did not suffer from sepsis and liver failure.

The patient's younger brother (II:3)showed a minimal gas in the stomach with no obvious gas shadows in the intestine using X-ray test, and was diagnosed with malrotation by UGI after birth (Figures 2E,F), and the length of the small intestine was 51 cm during the operation. After the operation, he was also managed similarly with continuous enteral nutrition (EN) supported by PN. His dependence for PN lasted for 40 days. Their parents were not close relatives. They were in good health and denied any history of exposure to poisons, drugs, or radiation. No abnormality was found during prenatal examination in both patients. At the last follow-up, the proband was 3 years and 10 months old, weighing 12.5 kg with a height of 96.5 cm corresponding to P3–P15 as per the 2006 WHO Child Growth Standards (17). Her diet is similar to that of a girl her age, and she can eat meat, vegetables and rice. Similarly, the younger brother was 14 months old, weighing 8.5 kg (P3–P15) with a height of 78 cm (P50). He is currently formula fed. When the siblings were evaluated for intelligence and motor behavior in the child care department, doctors found no significant neurodevelopmental deficits.

### Methods

This study fully complied with the Tenets of the Declaration of Helsinki, and was approved by the Ethics Committee of the Xiamen maternal and child health care hospital (Xiamen, Fujian, China). Informed consent was obtained from the subject's parents before testing.

#### Genomic DNA Extraction From Peripheral Blood

The peripheral venous blood sample (2 ml) was collected from children and parents using Qiagen's DNA Extraction Kit (Blood Genomic DNA Mini Kit) and the NanoDrop2000 spectrophotometer was used to detect the concentration and purity of DNA samples.

#### WES and Bioinformatics Analysis

The IDT XGen^®^ Exome Research Panel V1.0 capture probe was used for liquid hybridization with genomic DNA library sequence. DNA fragments in the target region were enriched to construct a full exon library. High-throughput sequencing (PE150) was performed using Illumina Novaseq 6000 series sequencers. The results were compared with the Human Genome Database (HGL9) using BWA software. Single nucleotide polymorphic sites, small deletions, and insertions were analyzed using GATK and VarScan software. DBSNP, HapMap, Exac, and 1000 genomic databases were used for filtering and screening. The mutation sites with frequency <0.05 were reserved. Polyphen-2 and SIFT software was used to predict the pathogenicity of suspicious mutations. Also, gene mutation-related databases HGMD and Clinvar were used to identify suspected pathogenic sites. Sequence mutation sites were interpreted according to the “Standards and Guidelines for Interpretation of Sequence Variations” issued by the American Society of Medical Genetics and Genomics. The related harmful mutations were screened based on the phenotype of the proband.

#### qPCR

Based on the results of high-throughput sequencing, qPCR was used to detect and verify the *CLMP* gene in the missing region of the children and their parents. The *CLMP* gene (NM_024769.5) sequence was obtained from the University of California, Santa Cruz. Proband and parents used different controls. Each sample made three repetitions and calculated the ratio according to the average value of CT value. The β-actin gene ACTB was used as the internal reference gene, and Primer 5.0 software was used to design primers for the *CLMP* gene. The length of the PCR product was 100–200 base pairs (bp) and the Tm value was about 60°C. The primers were produced by Shanghai Shenggong. Biological company synthesized and prepared the qPCR reaction system (Maxima^®^ SYBR Green qPCR Master Mix, no ROX), set the PCR reaction conditions, and used the Bio-Rad IQ5 qPCR instrument for amplification and analyses.

### Literature Review

The locations and types of the *CLMP* gene variant reported in the literature were searched on PubMed and summarized.

## Results

### WES

**Figure 3:**
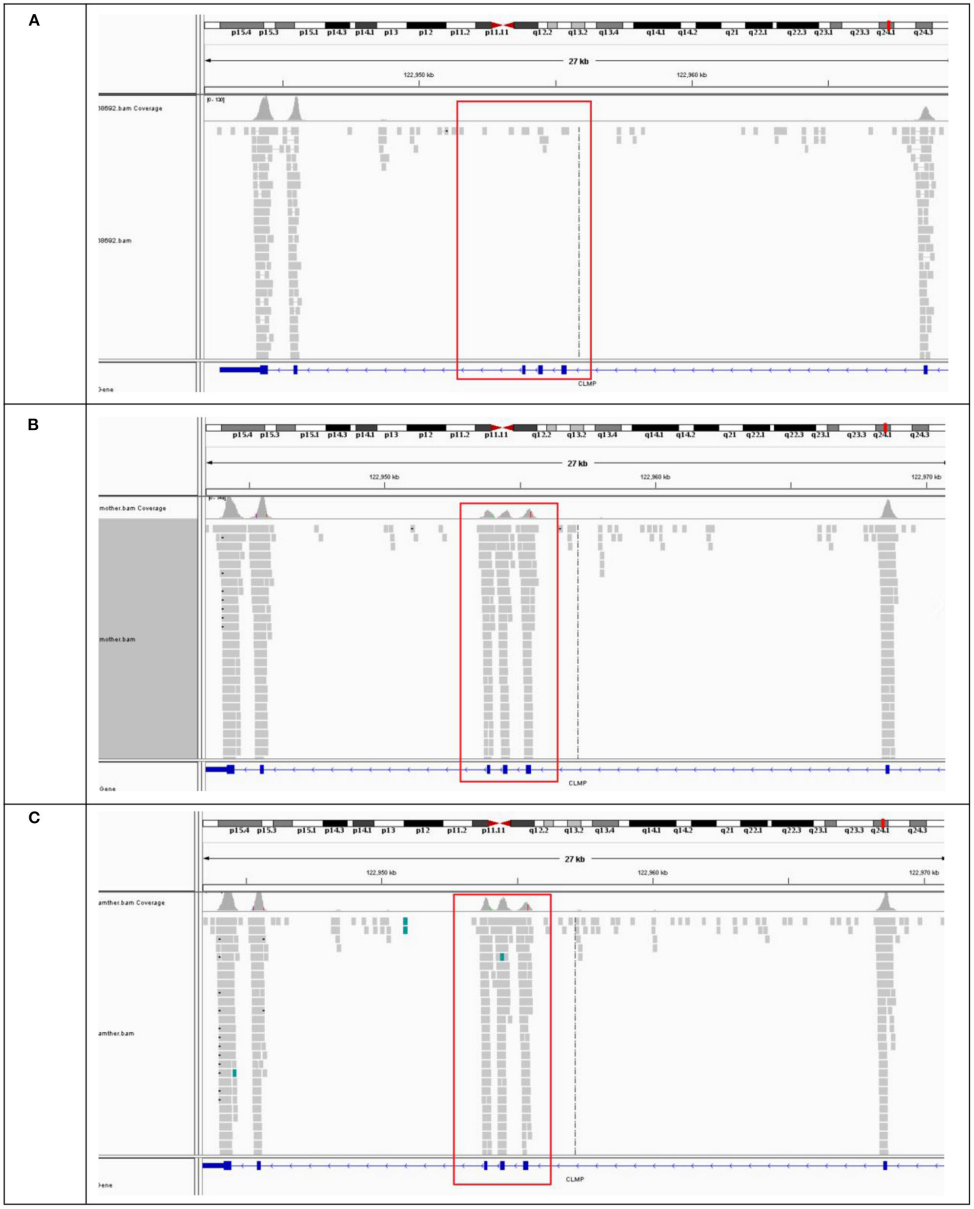
BAM map of *CLMP* gene sequenced in proband and family. **(A)** The BAM mape of the proband and **(B,C)** BAM mapes of proband's father and mother.

Analysis of the Trio-WES data revealed a homozygous deletion of 1,629 bp across exons 3 and 5 of the *CLMP* gene (CHR11:122953792-122955421) (Figure 3A) in proband. BAM diagram from IGV software showed that the proband's and her brother's *CLMP* gene had no coverage of exons 3 and 5 due to homozygous deletions (Figure 3A), and the father (Figure 3B) and mother (Figure 3C) were carriers of exon 3–5 deletions, so they had coverage of reads Trio-WES data confirmed homozygous the deletion of exons 3 and 5 of the proband *CLMP* gene. According to the classification standards of American College of Medical Genetics and Genomics (18), we found the a homozygous deletion of 1,629 bp across exons 3 and 5 of the *CLMP* gene was likely pathogenic pathogenis. While the variant is absent from a large general population harboring 1000 Genomes Project and Exome Aggregation Consortium (PM2).

### Analysis of CLMP by qPCR

**Figure 4:**
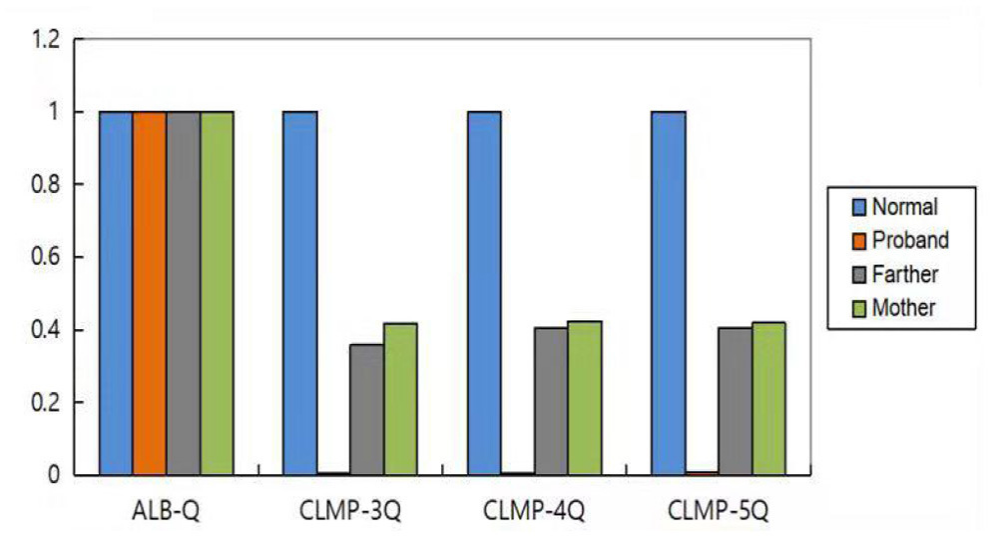
Results of exons 3–5 of the *CLMP* gene.

We used the *ALB* gene as the internal reference gene. qPCR was used to detect the number of copies of *CLMP* gene in the normal control samples and the samples from patients and their parents. The ratio of the number of copies of exons 3–5 of the *CLMP* gene to the normal control in the proband was nearly 0, and in the proband's parents was nearly 0.5. These results suggested that there was a homozygous deletion in exons 3–5 of the *CLMP* gene in the proband, and a heterozygous deletion in the parents (Figure 4).

## Discussion

CSBS refers to congenital shortening in the length of small intestine. It is a rare clinical condition. Wilmore *et al*. (25) proposed that full-term infants with the small intestinal length <75 cm are likely to have symptoms and signs of SBS. Only 74 cases have been reported since the first description by Hamilton in 1969 (8). Among the 74 cases, The age of patients at the time of diagnosis vary between 33 weeks of gestational age and 15 years old. The male-to-female ratio is 1.55 (45 males, 29 females). The main clinical manifestations of CSBS include biliary vomiting, abdominal distension, chronic diarrhea. Intestinal malrotation was identified in 70 cases. Most patients with CSBS have other abnormalities such as intestinal adhesions, intestinal atresia, pyloric hypertrophy, persistent patent ductus arteriosus, central nervous system malformations, common dorsal mesentery, absence of the appendix, and colon involvement. The diagnosis of CSBS mostly depends on early intestinal obstruction symptoms, imaging examination and surgical exploration.

In this study, we reported that two siblings in a family developed intestinal obstruction after birth, mainly manifested as vomiting. UGI and laparotomy found that both patients had intestinal shortening accompanied by malrotation. After genetic examination, homozygous deletion of exons 3–5 of *CLMP* gene was found in both siblings. There were heterozygous mutations in both parents, but no associated phenotype, suggesting an autosomal recessive inheritance of the disease. *CLMP* exons 3–5 encode amino acids at positions 63 to 227, which are located in two key domains of the protein (Ig-like C2-type 1, Ig-like C2-type 2). Through biological analyses, we found that the deletion of these amino acids can lead to the loss of protein function, thus predicting the pathogenicity.

According to literature review, a total of 18 children with CSBS have been confirmed to have *CLMP* mutations by genetic tests so far (Table 1). All *CLMP* gene mutations reported in the literature can lead to the loss of protein function, and the clinical manifestations of the reported cases are limited to the intestines. Summarizing these cases, no specific mutation types were found to be associated with specific clinical phenotypes.

**Table 1:** Literature review of CLMP gene variant in patients with CSBS.

**Reference**	**Pathogenic variant**	**Bowel length (cm)**
Huysman (19)	deletion of 12,483 bp including exon 2	54
De Backer (20)	410G>A (exon 4); p. (C137Y) c.29-2 A>G (exon 2)	50
Schalamon (3)	del (with presumed inversion) in intron 1	47
Ordonez (7)	c.230delA (exon 3); p (E77Gfs*24) c.821G >A (exon6)	30
Hasosah (5)	c.666C>T (exon 5); p. (R222X)	50
Van der Werf (10)	c.666C>T (exon 5); p. (R222X)	N/A
Van der Werf (10)	del (with presumed inversion)in intron 1	N/A
Van der Werf (10)	c.371T>A (exon 2); p.V124D	N/A
Alves (21)	c.508C>T (exon 4); p.(R170*)	26
Alves (21)	c.508C>T (exon 4); p.(R170*)	76
Gonnaud (22)	c.28 +1G > C (IVS1), c502C>T (exon 4); p. (R168X)	35
Gharesouran (23)	c.664C > T (exon 5); p. (R222X)	70
Wang (15)	c.206G>A, p.R69H	40
Wang (15)	c.206G>A, p.R69H	40
Wang (15)	c.206G>A, p.R69H	40
Wang (15)	C.655T>G, p.Cys219 Gly;C.389-2A>C	50
Chuang (24)	deletion across exon 3–5; c.235C > T, p.Q79* (exon 3)	30
Chuang (24)	deletion across exon 3-5; c.235C > T, p.Q79* (exon 3)	70

Langhorst *et al*. (26) studied *CLMP*-deficient mouse models and found that the mutant mice had malrotation of the intestine but no significant change in the length of the intestine compared to wild-type mice. The lack of *CLMP* gene can lead to a decrease in intestinal smooth muscle cells and ureteral junction protein 43, causing impaired intestinal, ureteral motor function, and functional obstruction. Thus, in addition to impaired intestinal motility, *CLMP*-deficient mice also have severe bilateral hydronephrosis. Alves *et al*. (21) described two patients with *CLMP* mutation [C.508C>T (Exon 4); P. (R170^*^)] and found that besides reduced intestinal length, the patients also had an obstruction of the ureteropelvic junction, similar to the mouse model. However, the same changes were not seen in other cases. Therefore, how *CLMP* variant types are associated with clinical phenotypes still needs further study.

To date, there is no cure for CSBS. The management of CSBS includes the maintenance of growth and development, promotion of intestinal adaptation, and prevention of complications (27). In the early stage of intestinal adaptation (1–3 months), it is necessary to maintain fluid and acid-base balance (28). The use of parenteral nutrition (PN) is inevitable and indispensable, but the long-term use of PN is often accompanied by several complications such as liver failure and sepsis (29), which are the most common causes of death in these patients. Negri *et al*. (30) noted in their study that the mortality of CSBS was 60.6%, and the main cause of death was malnutrition and/or sepsis. The survival rate was 30.6% before 2008 and 80.4% after 2008. The survival rate of children with CSBS has improved due to better nutritional management practices. For children with CSBS, early EN is of great importance to promote intestinal adaptation. In the early stage, the selection of milk and feeding methods needs to be continuously improved according to the condition of child until the intestinal tolerance is established in order to improve the prognosis.

## Conclusion

CSBS is one of the rare neonatal diseases. The loss of *CLMP* function is one of possible pathogenetic mechanisms for its development. CSBS is diagnosed by UGI, laparotomy, and genetic testing. These children require early EN with PN support to reduce complications and mortality. At present, the specific role of *CLMP* in the development of small intestine is still unclear, and further studies on the pathogenic mechanism of *CLMP* gene are needed. Parents of a child with CSBS with *CLMP* gene mutations can seek prenatal diagnosis for early detection and intervention in subsequent pregnancies.

## Data Availability Statement

The datasets for this article are not publicly available due to the Regulations of the People's Republic of China on the Administration of Human Genetic Resources in 2019. Requests to access the datasets should be directed to the corresponding authors (Xin-Zhu Lin, xinzhufj@163.com; Yan-an Wu, wyaslyy@126.com).

## Ethics Statement

The studies involving human participants were reviewed and approved by Ethics Committee of Maternal and Child Health Hospital of Xiamen city. Written informed consent to participate in this study was provided by the participants' legal guardian/next of kin.

## Author Contributions

F-fO and M-jL: responsible for article writing and genetic testing. L-bM: help with text revision and genetic interpretation. X-ZL and Y-aW: provide writing ideas and guidance. All authors contributed to the article and approved the submitted version.

## Funding

This work was supported by Young Investigator Research Program of Xiang'an Hospital of Xiamen University, China (No. PM202103050004).

## Conflict of Interest

The authors declare that the research was conducted in the absence of any commercial or financial relationships that could be construed as a potential conflict of interest.

## Publisher's Note

All claims expressed in this article are solely those of the authors and do not necessarily represent those of their affiliated organizations, or those of the publisher, the editors and the reviewers. Any product that may be evaluated in this article, or claim that may be made by its manufacturer, is not guaranteed or endorsed by the publisher.

## References

[1] Siebert JR. Small-intestine length in infants and children. Am J DisChild. (1980) 134:593–5. 10.1001/archpedi.1980.02130180051015 7386434

[2] Reiquam CW Allen RP Akers DR. Normal and abnormal smallbowel lengths: an analysis of 389 autopsy cases in infants andchildren. Am J Dis Child. (1965) 109:447–51. 10.1001/archpedi.1965.02090020449013 14280142

[3] Schalamon J Schober PH Gallippi P Matthyssens L Hollwarth ME. Congenital short-bowel; a case study and review of the literature. Eur J Pediatr Surg. (1999) 9:248–50. 10.1055/s-2008-1072255 10532268

[4] Orphanet. Congenital Short Bowel Syndrome. ORPHA:2301.

[5] Hasosah M Lemberg D Skarsgard E Schreiber R. Congenital shortbowel syndrome: a case report and review of the literature. Can J Gastroenterol. (2008) 22:714. 10.1155/2008/590143 18209785 PMC2659124

[6] Erez I Reish O Kovalivker M Lazar L Raz A Katz S. Congenital short-bowel and malrotation: clinical presentation and outcome of six affected offspring in three related families. Eur J Pediatr Surg. (2001) 11:331–4. 10.1055/s-2001-18546 11719873

[7] Ordonez P Sondheimer JM Fidanza S Wilkening G HoffenbergEJ. Long-term outcome of a patient with congenital short bowel syndrome. J Pediatr Gastroenterol Nutr. (2006) 42:576–80. 10.1097/01.mpg.0000189360.84169.da 16707984

[8] Hamilton JR Reilly BJ Morecki Morecki R: Short small intestine associated with malrotation:a newly described congenital cause of intestinal malabsorption. Gastroenterology. (1969) 56:124–36. 10.1016/S0016-5085(69)80074-0 5765427

[9] Tanner MS Smith B Lloyd JK. Functional intestinal obstruction due to deficiency of argyrophil neurones in the myenteric plexus. Familial syndrome presenting with short small bowel, malrotation, and pyloric hypertropy. Arch Dis Child. (1976) 51:837–41. 10.1136/adc.51.11.837 1008589 PMC1546064

[10] Van Der Werf CS Wabbersen T Paredes J Etchevers HC Kroisel PM. *CLMP* is required for intestinal development, and loss-of-function mutations cause congenital short-bowel syndrome. Gastroenterology. (2012) 142:453–62. 10.1053/j.gastro.2011.11.038 22155368

[11] Raschperger E Engstrom U Pettersson RF Fuxe J. *CLMP*, a novel member of the CTX family and a new component of epithelial tight junctions. J Biol Chem. (2004) 279:796–804. 10.1074/jbc.M308249200 14573622

[12] Sze KL Lui WY Lee WM. Post-transcriptional regulation of *CLMP* mRNA is controlled by tristetraprolin in response to TNFalpha via c-Jun N-terminal kinase signalling. Biochem J. (2008) 410:575–83. 10.1042/BJ20070901 18047469

[13] Balda MS Matter K. The tight junction protein ZO-1 and an interacting transcription factor regulate ErbB-2 expression. EMBO J. (2000) 19:2024–33. 10.1093/emboj/19.9.2024 10790369 PMC305688

[14] Matter K Balda MS. Signalling to and from tight junctions. Nat RevMol Cell Biol. (2003) 4:225–366. 10.1038/nrm1055 12612641

[15] Wang Y Chen S Yan W. Congenital short bowel syndrome: clinical and genetic presentation in China. JPEN J Parenter Enteral Nutr. (2020) 45:1009–15. 10.1002/jpen.1974 33464596

[16] Van der Werf CS Sribudiani Y Verheij JBGM Carroll M O'Loughlin E Chen CH. Congenital short bowel syndrome as the presenting symptom in male patients with *FLNA* mutations. Genet Med. (2013) 15:310–3. 10.1038/gim.2012.123 23037936

[17] World Health Organization. WHO Child Growth Standards: Length/Height-for-Age, Weight-for-Age, Weightfor-Length, Weight-for-Height and Body Mass Index-forAge: Methods and Development. Geneva: WHO (2006).

[18] Richards S Aziz N Bale S Bick D Das S Gastier-Foster J. Standards and guidelines for the interpretation of sequence variants: a joint consensus recommendation of the American College of Medical Genetics and Genomics and the Association for Molecular Pathology. Genet Med. (2015) 17:405–24. 10.1038/gim.2015.30 25741868 PMC4544753

[19] Huysman WA Tibboel D Bergmeijer JH Molenaar JC. Long-term survival of a patient with congenital short bowel and malrotation. J Pediatr Surg. (1991) 26:103–5. 10.1016/0022-3468(91)90442-V 2005514

[20] De Backer AI Parizel PM De Schepper A Vaneerdeweg W. A patient with congenital short small bowel associated with malrotation. J Belge Radiol. (1997) 80:71–2. 9237417

[21] Alves MM Halim D Maroofifian R de Graaf BM Rooman R van der Werf CS. Genetic screening of congenital short bowel syndrome patients confifirms *CLMP* as the major gene involved in the recessive form of this disorder. Eur J Hum Genet. (2016) 24:1627–9. 10.1038/ejhg.2016.58 27352967 PMC5055811

[22] Gonnaud L Alves MM Cremillieux C Billiemaz K Destombe S Varlet F. Two new mutations of the *CLMP* gene identifified in a newborn presenting congenital short-bowel syndrome. Clin Res Hepatol Gastroenterol. (2016) 40:e65–7. 10.1016/j.clinre.2015.12.018 27720179

[23] Gharesouran J Esfahani BS Valilou SF Moradi M Mousavi MH Rezazadeh M. First report of congenital short bowel syndrome in an iranian patient caused by a mutation in the *CLMP* gene. J Pediatr Genet. (2019) 8:73–80. 10.1055/s-0038-1675339 31061750 PMC6499612

[24] Chuang YH Fan WL Chu YD Liang K-H Yeh Y-M Chen C-C. Whole-exome sequencing identified novel *CLMP* mutations in a family with congenital short bowel syndrome presenting differently in two Probands. Front Genet. (2020) 11:574943. 10.3389/fgene.2020.574943 33384711 PMC7770137

[25] Wilmore DW. Factors correlating with a successful outcome following extensive intestinal resection in newborn infants. J Pediatr. (1972) 80:88–95. 10.1016/S0022-3476(72)80459-1 4552656

[26] Langhorst H Jüttner R Groneberg D Mohtashamdolatshahi A Pelz L Purfürst B. The IgCAM *CLMP* regulates expression of Connexin43 and Connexin45 in intestinal and ureteral smooth muscle contraction in mice. Dis Model Mech. (2018) 11:dmm032128. 10.1242/dmm.032128 29361518 PMC5894946

[27] Batra A Beattie RM. Management of short bowel syndrome in infancy. Early Hum Dev. (2013) 89:899–904. 10.1016/j.earlhumdev.2013.09.001 24125822

[28] Kumpf VJ. Pharmacologic management of diarrhea in patients with short bowel syndrome. J Parenter Enteral Nutr. (2014) 38:38S−44S. 10.1177/0148607113520618 24463352

[29] Sansaricq C Chen WJ Manka M Davis D Snyderman S. Familial congenital shortsmall bowel with associated defects. a long-term survival. Clin Pediatr. (1984) 23:453–5. 10.1177/000992288402300809 6428792

[30] Negri E Coletta R Morabito A. Congenital short bowel syndrome: systematic review of a rare condition. J Pediatr Surg. (2020) 55(9):1809–14. 10.1016/j.jpedsurg.2020.03.009 32278545

